# Tryptophan metabolism in digestive system tumors: unraveling the pathways and implications

**DOI:** 10.1186/s12964-024-01552-7

**Published:** 2024-03-11

**Authors:** Liang Yu, Juan Lu, Weibo Du

**Affiliations:** State Key Laboratory for Diagnosis, Treatment of Infectious Diseases,, Collaborative Innovation Center for Diagnosis and Treatment of Infectious Diseases, National Clinical Research Center for Infectious Diseases, National Medical Center for Infectious Diseases, The First Affiliated Hospital, Zhejiang University School of Medicine, No. 79 Qingchun Road, Shangcheng District, Hangzhou, Zhejiang 310003 China

**Keywords:** Metabolic reprogramming, Tryptophan metabolism, Biomarker, Mechanism, Treatment strategy

## Abstract

Tryptophan (Trp) metabolism plays a crucial role in influencing the development of digestive system tumors. Dysregulation of Trp and its metabolites has been identified in various digestive system cancers, including esophageal, gastric, liver, colorectal, and pancreatic cancers. Aberrantly expressed Trp metabolites are associated with diverse clinical features in digestive system tumors. Moreover, the levels of these metabolites can serve as prognostic indicators and predictors of recurrence risk in patients with digestive system tumors. Trp metabolites exert their influence on tumor growth and metastasis through multiple mechanisms, including immune evasion, angiogenesis promotion, and drug resistance enhancement. Suppressing the expression of key enzymes in Trp metabolism can reduce the accumulation of these metabolites, effectively impacting their role in the promotion of tumor progression and metastasis. Strategies targeting Trp metabolism through specific enzyme inhibitors or tailored drugs exhibit considerable promise in enhancing therapeutic outcomes for digestive system tumors. In addition, integrating these approaches with immunotherapy holds the potential to further enhance treatment efficacy.

## Background

Digestive system tumors rank among the most prevalent malignancies and remain a leading cause of cancer-related mortality, despite advancements in surgery, chemoradiotherapy, and immunotherapy [1–4]. The challenges lie in their elusive early detection and high invasiveness, particularly evident in liver and pancreatic cancers. Addressing this challenge requires focused efforts on identifying precise diagnostic biomarkers and effective therapeutic targets.

Metabolic reprogramming stands out as a hallmark of cancer, altering how tumor cells use energy and nutrients [5–7]. While normal cells primarily rely on oxidative phosphorylation for energy production, cancer cells favor anaerobic glycolysis. In addition, tumor cells exhibit an augmented demand for biosynthetic pathways, including amino acids, lipids, and nucleotides [8–10]. Trp metabolism emerges as a novel frontier in tumor metabolism research, given its role as both a component in protein synthesis and a precursor to biologically active molecules [11–13]. Beyond its physiological regulation in the human body, Trp and its metabolites play a crucial role in tumorigenesis and progression [13]. Differentially expressed Trp metabolites serve as potential diagnostic markers for tumors, while tumor cells use Trp to synthesize bioactive molecules influencing cell biological functions through signaling pathways or direct action [14, 15]. Trp metabolites further impact immune cells and the tumor microenvironment, exerting influence on tumor development [16, 17]. Investigating Trp metabolism enhances our comprehension of tumor genesis and development, presenting potential targets for innovative therapeutic strategies.

As a vital biological pathway, Trp metabolism significantly contributes to the onset and progression of digestive system tumors. Studies indicate that heightened Trp metabolism in digestive system tumor cells leads metabolite accumulation, affecting tumor growth, metastasis, and immune evasion [18, 19]. Trp degradation products modulate immune cell function, influencing the immune response to digestive system tumors. In addition, Trp metabolism regulates immune cell function, affecting the immune response to digestive system tumors. In-depth exploration of Trp metabolism has led to novel therapeutic strategies, including inhibiting key enzymes such as indoleamine 2,3-dioxygenase (IDO) and Trp 2,3-dioxygenase (TDO) to block Trp utilization, thereby enhancing immune response and inhibiting tumor growth. Combining IDO1 inhibitors with immune checkpoint inhibitors shows promise in improving treatment efficacy. Ongoing drug development targets the interference of Trp transport and utilization by tumor cells, underscoring the therapeutic potential of Trp metabolism in digestive system tumors. This review comprehensively summarizes the expression of Trp and its metabolites in digestive system tumors, elucidating their correlation with clinical features. In addition, it delves into the roles and mechanisms of Trp metabolism in the development of digestive system tumors. Finally, it introduces therapeutic strategies targeting Trp metabolism, underscoring their potential in advancing the treatment landscape for digestive system tumors.

### Overview of Trp metabolism

**Fig. 1:**
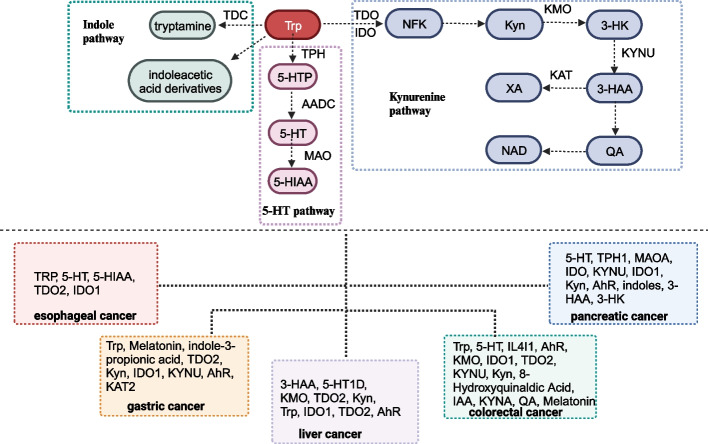
Aberrant Trp metabolism in digestive system tumors

Trp assumes vital physiological roles within the human body, serving as an essential amino acid for protein synthesis [20]. Beyond its structural role, it acts as a precursor to the neurotransmitter serotonin (5-HT), influencing mood regulation, sleep, and appetite control. Trp metabolism encompasses the intricate transformation and utilization of Trp, involving several pathways and key enzymes that regulate crucial physiological functions [21]. The three primary metabolic pathways of Trp metabolism are the kynurenine, 5-HT, and indole pathways (Fig. 1).

### Kynurenine pathway

The kynurenine pathway stands as the primary route in Trp metabolism, exerting pivotal roles in immune regulation, neurodevelopment, and brain function [14, 22–24]. Initiated by either TDO or IDO, Trp undergoes catalysis to form N-formyl-L-kynurenine (NFK). Subsequently, NFK is converted to kynurenine (Kyn), with kynurenine 3-monooxygenase (KMO) catalyzing the transformation of Kyn into 3-hydroxy-kynurenine (3-HK). The enzyme kynureninase (KYNU) facilitates the conversion of 3-HK into 3-hydroxyanthranilic acid (3-HAA). Further in the pathway, the enzyme kynurenine aminotransferase (KAT) transforms 3-HK into xanthurenic acid (XA). Notably, 3-HAA undergoes non-enzymatic degradation, resulting in the formation of quinolinic acid (QA), ultimately contributing to the synthesis of nicotinamide adenine dinucleotide (NAD) [24]. Crucially, IDO, TDO, and KMO serve as rate-limiting enzymes in the kynurenine pathway, exerting key regulatory roles in metabolic processes [25–27]. IDO, widely distributed in various cell types, including immune and non-immune cells, is subject to regulation by factors such as cytokines, inflammation, and immune responses. TDO, predominantly located in the liver, regulates the hepatic conversion of Trp [28]. Meanwhile, KMO, expressed across diverse cell types, participates in immune regulation and oxidative stress.

### 5-hydroxytryptamine pathway

5-hydroxytryptamine (5-HT, serotonin) is a versatile and ancient bioamine with diverse functions [29, 30]. Comprising integral components such as the serotonin transporter (SLC6A4), serotonin receptors (HTR), Trp hydroxylase (TPH), and monoamine oxidase, the 5-HT pathway plays a pivotal role in the Trp hydroxylation pathway. The synthesis of 5-HT, a neurotransmitter that regulates emotions, sleep, appetite, and other physiological functions, begins with Trp hydroxylation [29, 31]. This initial step is catalyzed by TPH, which converts Trp into 5-hydroxytryptophan (5-HTP). The conversion of 5-HTP to 5-HT requires L-aromatic amino acid decarboxylase (AADC) and cofactors like vitamin B6 [32]. 5-HT binds to various subtypes of 5-HT receptors on targeted cells, including 5-HT1, 5-HT2, and 5-HT3, eliciting diverse physiological effects in different tissues and organs. Monoamine oxidase (MAO) facilitates the metabolism of 5-HT into 5-hydroxyindoleacetic acid (5-HIAA), which is excreted by the kidneys. The 5-HT pathway exerts a crucial regulatory role in tissues and organs such as the digestive system, cardiovascular system, and immune system [33, 34]. Within the digestive system, 5-HT regulates gastrointestinal peristalsis and secretion. In the cardiovascular system, it participates in the regulation of blood pressure and vasoconstriction. In the immune system, 5-HT plays a role in modulating the function of immune cells.

### Indole pathway

The indole pathway constitutes a segment of the Trp decarboxylation (TDC) pathway, which is catalyzed by gut microbes. TDC catalyzes the conversion of Trp into tryptamine, a process mediated by intestinal microorganisms. Tryptamine undergoes further metabolism, generating various indoleacetic acid derivatives, including indoleacrylic acid, indole-3-acetic acid (IAA), and indole-3-acetaldehyde. Indole and its derivatives act as natural ligands for the aryl hydrocarbon receptor (AhR), activating AhR by binding to it and thereby regulating the transcription of downstream genes. Activated AhR–ligand complexes play a crucial role in regulating diverse biological processes such as detoxification metabolism, immune regulation, and development. AhR promotes the expression of detoxification enzymes, aiding the body in eliminating harmful substances. In addition, AhR regulates the differentiation and function of immune cells, actively participating in immune response regulation. Various substances, including polycyclic aromatic compounds, polychlorinated biphenyls, and Kyn, can influence AhR activity. As a ligand-activated transcription factor, AhR plays a pivotal regulatory role in fundamental biological processes.

### Role of Trp mechanism in digestive system tumors

#### Esophageal cancer (EC)

In patients with EC, serum Trp levels are significantly reduced compared to healthy controls, while serum serotonin (5-HT) and 5-HIAA levels are notably elevated in patients with EC compared to healthy controls (Fig. 1) (Table 1) [35, 36]. Overexpression of IDO2 is observed in ESCC tissues [37, 38]. Elevated 5-HT emerges as a crucial indicator of EC susceptibility (AUC = 0.811), and increased plasma 5-HIAA is strongly associated with a higher likelihood of lymph node metastasis [36]. A higher XA/Kyn ratio is linked to shorter overall survival. Notably, XA/Kyn levels are significantly correlated with lower overall survival in patients with EC [36]. Increased TDO2 levels are significantly associated with lymph node metastasis, advanced clinical stage, recurrence status, and unfavorable prognosis in patients with EC [37, 38]. CD44, a commonly used tumor stem cell marker in EC, can self-renew and rebuild tumor tissues and is notably expressed in ESCCs with high TDO2 expression, indicating its potential role in tumor recurrence, metastasis, and drug resistance [38]. Patients with negative IDO1 expression exhibit significantly improved overall survival compared to those with positive IDO1 expression [39]. High CD8 expression is associated with better overall survival and lower IDO1 expression. Consistently upregulated ratios of Kyn/Trp, 5HTP/Trp, 5HIAA/Trp, and 5HT/Trp in patients with ESCC emerge as promising biomarkers for tumorigenesis and metastasis [35]. Trp and its metabolites show potential as postoperative prognosis markers in patients with EC, with a sharp decrease in plasma total Trp concentration observed after surgery [40]. Functionally, TDO2 enhances tumor cell proliferation, migration, and colony formation, with inhibition of TDO2 preventing these effects [37, 38]. TDO2 overexpression contributes to in vivo tumor growth [37]. Mechanistically, TDO2 upregulates interleukin-8 (IL-8), activating the protein kinase B (AKT)/ glycogen synthase kinase-3 (GSK3β) pathway and promoting M2 macrophage polarization, thereby accelerating tumor progression in EC [37].

**Table 1:** Role and mechanism of tryptophan mechanism in digestive system tumors

**Tumor type**	**Tryptophan metabolism**	**Sample**	**Clinical characteristics**	**Functions**	**Mechanisms**	
Esophageal cancer	Trp, 5-HT	Serum	Metastasis			[35]
Esophageal cancer	5-HT, 5-HIAA	Serum	Susceptibility, lymph node metastasis, prognosis			[36]
Esophageal cancer	TDO2	Tissue	Lymph node metastasis, clinical stage, prognosis	Cell proliferation, migration, colony formation, tumor growth	AKT, GSK3Β, IL-8	[37]
Esophageal cancer	TDO2	Tissue	Tumor stage, recurrence status, prognosis	Cell proliferation, spheroid colonies, invasion	PI3K, AKT, ERK	[38]
Esophageal cancer	IDO1	Tissue	Prognosis			[39]
Gastric cancer	Trp, Melatonin, Indole-3-propionic acid	Serum				[41]
Gastric cancer	Trp	Serum	Sensitivity of predicting gastric cancer			[42]
Gastric cancer	TDO2	Tissue	TNM stage, lymphatic invasion status, prognosis			[43]
Gastric cancer	Kyn	Serum	Drug resistance	Chemoresistance	IL-10, STAT3, BCL2	[44]
Gastric cancer	Trp	Gastric juice	Detection of early gastric cancer			[45]
Gastric cancer	IDO1	Tissue		Parietal cell loss, gastric metaplasia	B cell	[46]
Gastric cancer	IDO1	Cell		Cell migration	MAPK pathway	[47]
Gastric cancer	KYNU, Kyn, AhR	Tissue		Metastasis	GPX2	[48]
Gastric cancer	KAT2		Gastric intestinal metaplasia		CGAS, IRF3	[49]
Liver cancer	3-HAA	Tissue		Cell apoptosis, tumor growth	YY1, PKC, DUSP6, ERK1/2	[50]
Liver cancer	5-HT1D	Tissue	Recurrence rate, prognosis	Cell proliferation, epithelial-mesenchymal transition, metastasis	PI3K, AKT, FOXO6	[51]
Liver cancer	KMO	Tissue	Tumor differentiation, disease recurrence, prognosis	Cell proliferation, migration, invasion		[52]
Liver cancer	TDO2	Tissue	Tumor size, tumor differentiation, m stage, prognosis	Cell migration, invasion, proliferation	CircZNF566, mir-4738-3p	[53]
Liver cancer	Kyn, Trp	Serum	Increased risk of HCC			[54]
Liver cancer	IDO1	Tissue	Prognosis			[55]
Liver cancer	IDO1	Tissue	Prognosis			[56]
Liver cancer	TDO2	Tissue	Advanced stage, vascular invasion, prognosis	Cell migration, invasion		[57]
Liver cancer	Trp, Kyn, AhR	Tissue		Cell proliferation, migration	CYP1A1	[58]
Liver cancer	TDO2	Tissue	Tumor size, clinical stage, TNM stage, tumor differentiation, prognosis	Cell proliferation, cell-cycle arrest, tumor growth	P21, P27	[59]
Colorectal cancer	Trp	Serum				[60]
Colorectal cancer	5-HT	Tissue		Tumor growth	NLRP3, Immortalized bone marrow-derived macrophages	[61]
Colorectal cancer	IL4I1, AhR, Trp	Tissue		Cell proliferation, migration, invasion		[62]
Colorectal cancer	KMO	Tissue	Metastasis, prognosis	Sphere-forming, migration, invasion		[63]
Colorectal cancer	IDO1, TDO2	Tissue	Lymph node metastasis, tumor stage			[64]
Colorectal cancer	TDO2, KYNU, AhR	Tissue	Clinical stage, prognosis	Cell proliferation, colony formation, invasion, tumor growth		[65]
Colorectal cancer	Tryptophanyl-trna synthetase	Tissue	Lymph node metastasis, tumor stage, risk for recurrence, prognosis			[66]
Colorectal cancer	IDO1	Tissue		Cell proliferation, tumor growth	Β-catenin	[67]
Colorectal cancer	5-HT	Tissue		Angiogenesis, tumor growth	Macrophages	[68]
Colorectal cancer	Kyn, AhR	Cell		Goblet cell differentiation	WNT, NOTCH, HES1, HATH1	[69]
Colorectal cancer	IDO1	Tissue		Cell proliferation, apoptosis, tumor growth	PI3K, AKT	[70]
Colorectal cancer	TDO2, AhR, Kyn	Tissue	Liver metastasis	Stemness, immune evasion	PD-L1	[71]
Colorectal cancer	8-hydroxyquinaldic acid	Tissue		Cell proliferation, cell cycle, cell migration	Β-catenin, E-cadherin	[72]
Colorectal cancer	IAA	Cell		Cell proliferation, cell cycle	TLR4, ERK, JNK	[73]
Colorectal cancer	KYNA	Cell		Cell proliferation	PI3K, AKT, MAPK, Β-catenin	[74]
Colorectal cancer	QA	Cell		Cell proliferation	ERK, P38, CREB, AKT	[75]
Colorectal cancer	Melatonin	Cell		Cell proliferation, apoptosis, angiogenesis	FOXO-1, NF-ΚΒ, Endothelin-1	[76]
Pancreatic cancer	5-HT, TPH1, MAOA	Tissue	Tumor stage, tumor size, prognosis	Cell proliferation, apoptosis, tumor growth	MYC, HIF1A, PI3K, AKT, MTOR	[77]
Pancreatic cancer	IDO	Tissue	TNM stage, histological differentiation, lymph nodule metastasis			[78]
Pancreatic cancer	KYNU	Tissue	Prognosis		NFR2	[79]
Pancreatic cancer	IDO1, Kyn, AhR	Tissue	Prognosis	Spheroid growth, invasion	NO•, RUNX3	[80]
Pancreatic cancer	AhR, Indoles	Cell	Prognosis	Tumor growth	TAM	[81]
Pancreatic cancer	3-HAA, 3-HK	Serum	Increased risk of pancreatic cancer			[82]

#### Gastric cancer

**Fig. 2:**
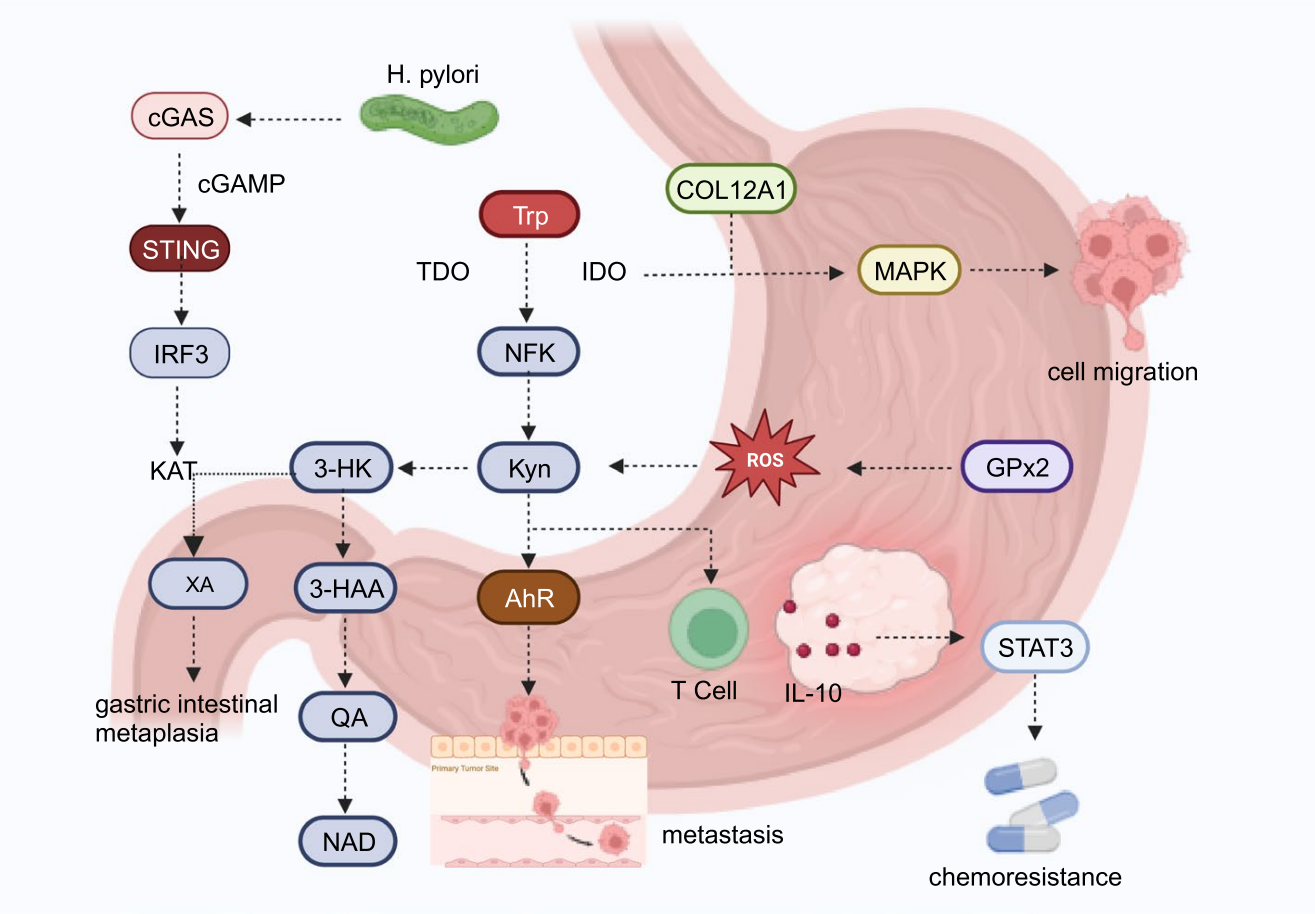
Molecular mechanisms of Trp metabolism in gastric cancer. *Helicobacter pylori* infection induced kynurenine pathway activation mediated by kynurenine aminotransferase 2 (KAT2) through cGAS-IRF3 signaling, fostering gastric intestinal metaplasia. IDO1 and COL12A1 exhibited a synergistic effect, enhancing cell migration via the MAPK pathway. GPx2 enhanced the KYNU-Kyn-AhR signaling pathway, promoting gastric cancer progression and metastasis. Kyn generated by gastric cancer cells activated the IL-10/STAT3/BCL2 pathway, excessively stimulating Tregs and crucially contributing to chemoresistance

In patients with gastric cancer, serum levels of Trp, melatonin, and indole-3-propionic acid are downregulated, while indole levels are increased [41, 42]. The expression of TDO2 in gastric cancer tissues and cells is significantly higher than that in the control group (Fig. 1) [43]. Trp and its metabolites exhibit associations with various clinical features in gastric cancer. Serum Kyn levels positively correlate with drug resistance in patients with gastric cancer [44]. TDO2 expression is positively correlated with T grade, N grade, M grade, and lymphatic invasion status [43]. In addition, TDO2 expression is negatively correlated with overall survival and is an independent predictor of gastric cancer prognosis [43]. Plasma-free Trp levels demonstrate higher sensitivity in predicting gastric cancer, and Trp levels in gastric juice serve as potential biomarkers for early detection [42, 45]. Functionally, TDO2 promotes cell proliferation, colony formation, invasive ability, and spheroid formation in gastric cancer. Knockdown of TDO2 significantly decreases the viability of gastric cancer organoids. IDO1 contributes to parietal cell loss and gastric metaplasia, acting as a critical mediator of immune suppression (Fig. 2) [46]. IDO inhibits T cell-mediated cytotoxicity and cell proliferation in gastric cancer, playing a crucial role in gastric metaplasia by modulating B-cell activity [46, 83]. IDO1 and COL12A1 synergistically enhance cell migration through a positive feedback loop mediated by the mitogen-activated protein kinases (MAPK) pathway [47]. Kyns derived from gastric cancer cells overstimulate regulatory T cells (Tregs) through the IL-10/ signal transducer and activator of transcription 3 (STAT3)/ B-cell lymphoma 2 (BCL2) signaling pathway, promoting chemoresistance [44]. Glutathione peroxidase 2 (GPx2) contributes to the progression and metastasis of gastric cancer by enhancing the KYNU-Kyn-AhR signaling pathway [48]. *Helicobacter pylori* facilitates gastric intestinal metaplasia by activating the kynurenine pathway mediated by KAT2 through the cyclic GMP-AMP synthase (cGAS)-Interferon regulatory factor 3 (IRF3) signaling [49].

#### Liver cancer

**Fig. 3:**
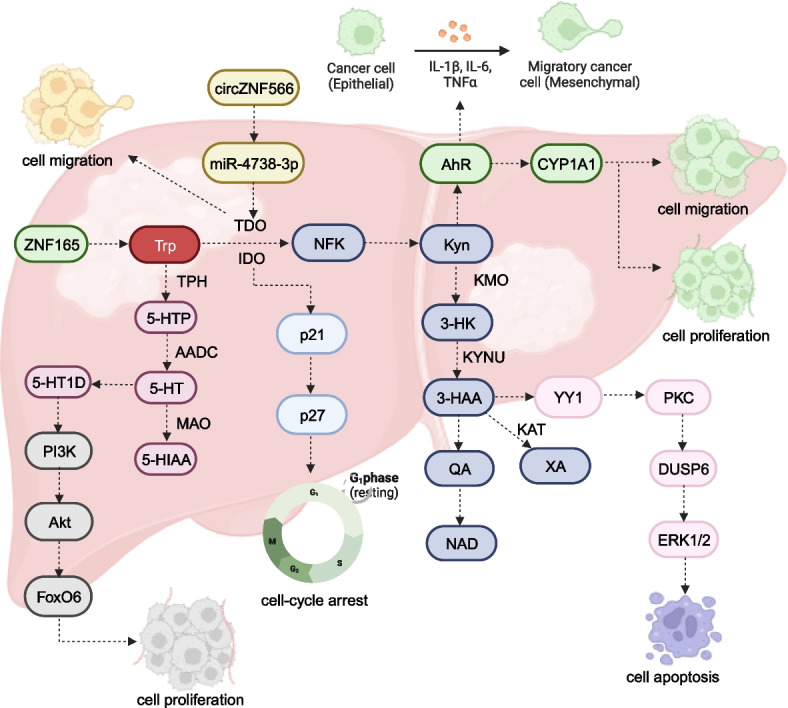
Molecular mechanisms of Trp metabolism in liver cancer. circZNF566 promoted HCC progression by sponging miR-4738-3p and modulating TDO2 expression. ZNF165 enhanced HCC cell proliferation and migration through robust activation of the Trp/Kyn/AhR/CYP1A1 axis, significantly increasing CYP1A1 expression. 5-HT1D, through PI3K/Akt/FoxO6 signaling, accelerated HCC progression. TDO2 upregulated p21 and p27 expression, exerting a suppressive impact on cell cycle arrest. The binding of 3-HAA to YY1 induced apoptosis in HCC cells

In hepatocellular carcinoma (HCC) tissues, 3-HAA levels are significantly downregulated, while expression of 5-HT1D, KMO, and TDO2 is markedly upregulated (Fig. 1) [50–52]. TDO2 overexpression correlates closely with HCC tumor size, degree of differentiation, and M phase, with higher TDO2 levels associated with worse overall survival and disease-free survival (DFS) [53]. KMO expression is correlated with tumor differentiation, and patients with HCC with elevated KMO expression exhibit decreased overall survival and a higher risk of disease recurrence [52]. Elevated 5-HT1D expression is linked to lower overall survival rates and increased recurrence rates in patients with HCC [51]. Higher levels of Kyn and Kyn/Trp ratio are associated with an elevated risk of HCC [54]. In addition, high IDO1 expression and increased infiltration of CD8 + T cells are significantly associated with overall survival in patients with HCC [55]. IDO1 expression serves as an independent prognostic factor for overall survival, DFS, and recurrence-free survival rates in HCC [55, 56]. kynurenine and kynurenine/ Trp ratio were associated with higher HCC risk. Functionally, TDO2 and KMO stimulate cell proliferation, migration, invasion, and epithelial-to-mesenchymal transition (EMT) in HCC cell lines [52, 53, 57]. Silencing TDO2 in vivo effectively suppresses intrahepatic tumor metastasis of liver cancer cells [57]. 5-HT1D markedly enhances cell proliferation, EMT, and metastasis in HCC [51]. Moreover, 3-HAA inhibits tumor growth and reduces tumor weight in vivo, triggering apoptosis in HCC cells by interacting with YY1 (Fig. 3) [50]. TDO2 stimulation facilitates the EMT process by activating the Kyn-AhR pathway [57]. 5-HT1D promotes HCC progression by activating the PI3K/Akt/FoxO6 signaling pathway [51]. circZNF566 promotes HCC progression by sponging miR-4738-3p and regulating TDO2 expression [53]. ZNF165 enhances the proliferation and migration of HCC cells by activating the Trp/Kyn/AhR/CYP1A1 axis and substantially enhancing CYP1A1 expression [58]. Interestingly, a study by Ding et al. showed differing views on the role of TDO2 in HCC. They found TDO2 downregulated in HCC tissues and its expression negatively correlated with tumor size, clinical stage, TNM stage, and tumor differentiation [59]. Lower TDO2 levels are associated with worse overall survival and DFS in patients with HCC. TDO2 inhibits cell proliferation by inducing cell cycle arrest in vitro and exerts an inhibitory effect on tumor growth and weight in vivo by upregulating the expression of p21 and p27 [59].

#### Colorectal cancer

In patients with colorectal cancer (CRC), serum Trp levels are significantly downregulated, with alterations in Trp metabolites and key enzymes (Fig. 1) [60]. The expression of IL4I1, KYNA, KMO, IDO1, TDO2, 5-HT, and TPH1 is notably increased in CRC tissues, with strong correlations observed between these TRP metabolites and various clinical features [61–65, 84]. Elevated IDO1, TDO2, and tryptophanyl-tRNA synthetase (TrpRS) expression predicts lymph node metastasis and advanced clinical stages in CRC [64, 66]. Furthermore, IDO1, TDO2, KYNA, and KMO expressions are positively associated with CRC metastasis, and higher levels of KMO, IDO1, TDO2, and TrpRS correspond to worse prognoses [63, 65, 71, 85, 86]. Upregulated TrpRS expression is negatively correlated with the risk of relapse in patients with CRC [66]. Functionally, IL4I1, KMO, TDO2, IDO1, and 5-HT expressions promote cell proliferation and invasion in CRC [62, 63, 65, 67, 68]. Trp deficiency attenuates the tumor-promoting effect of IL4I1 [62]. Kyn induces goblet cell differentiation, and 5-HT contributes to angiogenesis in CRC [68, 69]. In vivo experiments demonstrate the roles of TPH1 and IDO1 in promoting CRC growth [61, 68, 70]. Mechanically, 5-HT enhances the NLRP3 inflammasome activation in THP-1 cells and immortalized bone marrow-derived macrophages by interacting with the 5-hydroxytryptamine receptor 3A (HTR3A) receptor [61]. Silenced TDO2 reduces tumor growth by interacting with the KYNU-AhR pathway [65]. In addition, TDO2 suppresses the immune response and facilitates liver metastasis by inducing AhR-mediated PD-L1 trans-activation in CRC [71]. Kynurenine pathway metabolites, except kynurenic acid, enhance the malignant phenotype of CRC by activating the PI3K/AKT pathway [70]. Kyn and QA contribute to tumor growth via the activation of β-catenin [67].

Contrastingly, 8-hydroxyquinaldic acid, IAA, KYNA, melatonin, quinaldic acid, and KYNA attenuate CRC cell proliferation [72–75]. Melatonin inhibits cell proliferation by inducing the G2/M phase arrest [76]. IAA inhibits tumor progression by activating the Toll-like receptor 4 (TLR4)-c-Jun N-terminal kinase (JNK) pathway in CRC [73]. Melatonin significantly downregulates endothelin-1 levels, inhibiting CRC malignant phenotypes by reducing forkhead transcription factor-1 (FOXO-1) and nuclear factor-kappabeta (NF-κβ) levels [76].

#### Pancreatic cancer

In pancreatic cancer tissues, levels of 3-IAA, 5-HT, KYNU, IDO, and TPH1 are significantly elevated, while levels of MAOA, 3-hydroxyanthranilic acid 3,4-dioxygenase (HAAO), and quinolinic acid phosphoribosyltransferase (QPRT) are markedly downregulated (Fig. 1) [77–79, 87]. Upregulated IDO expression is negatively associated with TNM stage, histological differentiation, and lymph nodes metastasis in patients with pancreatic cancer [78]. TPH1 expression is positively correlated with TNM stage and tumor size, and IDO expression is elevated in pancreatic cancer metastases [88]. High levels of IDO, TPH1, HTR2B, AhR, and Kyn predict a poor prognosis in pancreatic cancer, whereas MAOA expression is negatively correlated with TNM stage and tumor size in pancreatic cancer, predicting a favorable prognosis [77, 78, 80, 81]. Serum 3-HAA levels and the HAA:3-HK ratio are negatively associated with the risk of pancreatic cancer [82]. Functionally, Kyn enhances spheroidal growth and cell invasion in pancreatic cancer [80]. 5-HT and HTR2B agonists promote cell proliferation and inhibit cell apoptosis in vitro, with silenced HTR2B blocking tumor growth in vivo, making it a potential drug target for pancreatic cancer treatment [77]. Tumor-associated macrophage (TAM) exhibits high AhR activity, and inhibiting AhR in TAM reduces pancreatic ductal adenocarcinoma (PDAC) growth [81]. Administration of Trp and 3-HAA increases sensitivity to chemotherapy drugs in vivo [87]. Mechanistically, the oxidation of 3-IAA combined with chemotherapy reduces antioxidant enzymes, leading to ROS accumulation and inhibition of autophagy in cancer cells, affecting cell metabolism and PDAC progression [87]. The conversion of Trp to indoles metabolized by lactic acid bacteria increases TAM AhR activity, recruiting TNF-α^+^IFN-γ^+^CD8^+^ T cells in PDAC, exerting an anti-tumor immune effect [81]. In addition, 5-HT enhances PDAC cell metabolism by upregulating key enzymes involved in glycolysis, pentose phosphate pathway, and hexosamine biosynthesis pathway [77]. Nitric oxide activates the IDO1/ kynuridine /AhR signaling axis, promoting pancreatic cancer progression [80].

### Application of Trp metabolism in digestive system tumor treatment

#### Targeting IDO1 and TDO in digestive system tumor treatment

IDO1 and TDO, pivotal enzymes in the kynurenine pathway, regulate the generation of various metabolites from Trp. Their involvement in promoting digestive system tumor progression through multiple mechanisms has identified them as potential therapeutic targets. Advances in IDO1 and TDO inhibitors have shown promise in clinical trials. These inhibitors inhibit tumor progression by blocking the activity of IDO1 and TDO through different mechanisms. Notable inhibitors include 1-MT (Table 2), a canonical IDO inhibitor with low toxicity, and its isomer 1-L-MT, which induces mitochondrial damage, reduces CRC cell proliferation, and inhibits CRC development in vivo [67, 89]. In addition, 1-D-MT inhibits CRC progression; however, its effect is weaker than that of 1-L-MT [89, 90]. The TDO inhibitor 680C91 induces the G2 phase arrest and apoptosis in CRC cell lines [91]. Sertaconazole nitrate dose-dependently inhibits IDO1 expression in CRC cells, promoting autophagy and apoptosis and inhibiting tumor growth [92]. Hydroxyamidine inhibitors show efficacy in CRC and pancreatic cancer by targeting IDO1 [93]. Hydrogen sulfide downregulates IDO1, increasing CD8^+^ T cells and inhibiting HCC progression [94]. Abrine inactivates the IDO1/JAK1/STAT1 axis, suppressing immune escape and HCC progression [95]. Carbidopa, an AhR agonist, inhibits IDO1 expression in PDAC cells, regulating the JAK/STAT pathway to impede pancreatic cancer progression [96]. However, unexpected outcomes in IDO1 inhibitor clinical trials may be attributed to AhR activation, impacting immunotherapy [97]. Notably, USP14 knockdown upregulates cytotoxic T-cell activity and enhances anti-tumor immunity in CRC by downregulating IDO1 expression without affecting AhR activity, suggesting a potential avenue for CRC-targeted therapy [98]. Sodium Tanshinone IIA Sulfonate (STS) inhibits both IDO1 and TDO2, restraining tumor growth and enhancing the anti-tumor activity of PD1 antibodies [99]. IDO1/TDO dual inhibitor RY103 blocks the kynurenine pathway in pancreatic cancer, inhibiting cell motility and demonstrating growth inhibition in vivo [100].

**Table 2:** Research on the application of trp metabolism in digestive system tumors

Targeted agents	Diseases	Molecules targeted	Outcomes	Ref
1-MT	Colorectal cancer	IDO	Inhibit cell proliferation, and tumor growth	[67]
1-MT	Pancreatic cancer	IDO	Motivate antitumor responses, and elicit tumor inflammatory necrosis	[90]
1-L-MT	Colorectal cancer	IDO	Inhibit cell proliferation, and tumor growth	[89]
Sertaconazole nitrate	Colorectal cancer	IDO1	Induce autophagy and apoptosis, and inhibit tumor growth	[92]
INCB023843, and INCB024360	Pancreatic cancer, and colorectal cancer	IDO	Inhibit the growth of IDO-expressing tumors	[93]
Hydrogen sulfide	Hepatocellular carcinoma	IDO1	Decrease microvasculature density, and enhance tumor cell apoptosis	[94]
Carbidopa	Pancreatic cancer	AHR, IDO1	Inhibit tumor growth	[96]
USP14	Colorectal cancer	IDO1	Promote immune suppression	[98]
Sodium tanshinone IIA sulfonate	Colorectal cancer	IDO1/TDO2	Enhance Anti-PD1 Therapy	[99]
RY103	Pancreatic cancer	IDO1/TDO	Inhibit cell migration, invasion, and tumor growth	[100]
GR127935	Colorectal cancer	5-HT1DR	Inhibit cell invasion, and tumor metastasis	[101]
Sb204741	Pancreatic cancer	HTR2B	Inhibit tumor growth	[77]
PF06845102/EOS200809	Colorectal cancer	TDO	Improve the efficacy of checkpoint inhibitors	[102]
Combination of Navoximod, and Atezolizumab	Pancreatic cancer	IDO1	Not offer convincing evidence of enhancement compared to single-agent therapy	[97]
Combination of Abrine, and Anti-PD-1 antibody	Hepatocellular carcinoma	IDO1	Reduce immune escape	[95]
Combination of 1-MT, and Radiation	Colorectal cancer	IDO	Enhance radiosensitivity	[103]
Combination of Rosmarinic acid, and IDO1-shRNA	Hepatocellular carcinoma	IDO	Inhibit tumor growth	[104]

#### Targeting KMO in digestive system tumor treatment

KMO, a crucial enzyme in Trp metabolism, represents a rate-limiting step in this pathway and is implicated in the development of various digestive system tumors. Modulating Trp metabolism by inhibiting KMO expression offers a potential avenue for impacting tumor growth and immune response. Slower research progress is observed on KMO inhibitors compared to IDO/TDO inhibitors, possibly due to the complex role and regulatory mechanisms of KMO, requiring further research to understand its specific role in digestive system tumors. However, recent studies underscore KMO as a promising therapeutic target in digestive system tumors. KMO upregulation is significantly observed in liver cancer and CRC, correlating with worsened patient prognosis in these malignancies [52, 63]. In HCC cell lines, KMO demonstrates a significant enhancing effect on cell proliferation, migration, invasion, and EMT [52]. Similarly, KMO aggravates the malignant phenotype of tumors in CRC [63]. KMO knockdown shows promise in inhibiting cancer progression by modulating cell biological functions in HCC and CRC.

#### Targeting TPH in digestive system tumor treatment

TPH, a pivotal enzyme in the 5-HT pathway, catalyzes the conversion of Trp to 5-HTP, influencing the synthesis and release of 5-HT. The potential involvement of the 5-HT pathway in digestive system tumor progression highlights TPH as a key therapeutic target. In CRC, 5-HT, through interaction with the HTR3A receptor, promotes tumor progression by activating the NLRP3 inflammasome. In vivo experiments demonstrate that inhibiting TPH1 with 4-chloro-DL-phenylalanine or using the HTR3A antagonist tropisetron reverses this effect [61]. In PDAC, 5-HT and HTR2B agonists promote cell proliferation while inhibiting cell apoptosis. The HTR2B antagonist SB204741 impedes tumor growth and enhances prognosis in PDAC [77]. In addition, 5-HT knockdown significantly inhibits angiogenesis in CRC [68]. Notably, a 5-HT(1D)R antagonist GR127935 suppresses tumor metastasis by regulating the Axin1/β-catenin/MMP-7 axis (Table 2) [101]. However, the role of 5-HT in CRC development remains controversial. Selective serotonin reuptake inhibitors decrease CRC risk by enhancing 5-HT expression [105]. Kannen et al. found that 5-HT can reduce DNA damage and CRC risk in the early stages of CRC, but its contribution to tumor growth in CRC complicates its overall impact [106]. This controversy underscores the need for further research to comprehensively understand the mechanisms of 5-HT and establish a theoretical basis for relevant therapies.

#### Combining Trp metabolism targeting with other therapies in digestive system tumor treatment

As tumor treatment strategies evolve, including surgical resection, chemotherapy, radiation therapy, targeted therapy, immunotherapy, and hormone therapy, challenges like drug resistance, recurrence, and metastasis persist. Targeting Trp metabolism has gained attention, but its adverse effects on normal physiological processes necessitate further research for safe and effective strategies. Combining targeted Trp metabolism with other therapies emerges as a promising avenue. In liver cancer, PD-1/PD-L1 monoclonal antibody therapy has shown promise by targeting immune checkpoint molecules [95]. Combining Abrine and anti-PD-1 antibodies synergistically inhibits HCC tumor growth [95]. TDO inhibitors enhance the efficacy of immune checkpoint inhibitors [102]. The dual IDO1/TDO2 inhibitor, STS, effectively boosts the anti-tumor effects of the PD1 antibody in CRC [99]. Although Phase I clinical trials did not confirm enhanced benefits, further research, possibly focusing on patients with positive IDO tumors, is warranted [97]. CAR T cell therapy, an emerging immunotherapy, shows potential in treating CRC [107]. In vivo studies have found that miR-153 enhances CAR T cell immunotherapy by inhibiting IDO1 expression in CRC. Moreover, 1-MT reduces radiation resistance in CRC cell lines, and its combination with radiation significantly inhibits CRC growth compared to monotherapy [103]. In HCC, combining rosmarinic acid with IDO1-shRNA demonstrates inhibitory effects on tumor progression in vivo [104].

## Conclusion

Trp, an essential amino acid, plays vital roles in physiological processes, serving as a precursor for molecules such as serotonin, melatonin, and niacin (Vitamin B3). Its metabolism is a critical pathway and significantly influences the progression of digestive system tumors. Elevated Trp metabolism leads to the accumulation of Trp metabolites, impacting malignant phenotypes across ESCC, gastric cancer, liver cancer, CRC, and pancreatic cancer. Aberrantly expressed Trp metabolites correlate with clinical features such as lymph node metastasis, TNM stage, risk of relapse, and tumor size in digestive system tumors. Furthermore, these metabolites closely tie to patients’ prognosis, showcasing diagnostic potential. Detecting Trp metabolite levels in blood, urine, or tissue samples provides clinicians with valuable insights for early detection, diagnosis, and prognostic assessment. A multi-parameter diagnostic model, integrating Trp metabolite expressions and other clinical indicators, improves diagnostic accuracy, which is crucial for early detection, personalized treatment planning, and treatment response monitoring in digestive system tumors. In digestive system tumors, Trp metabolites contribute to progression, metastasis, and drug resistance through diverse mechanisms. Inhibiting key enzymes in Trp metabolism can reduce metabolite accumulation, impeding their tumor-promoting effects. Adopting a comprehensive treatment strategy involves combining drugs targeting Trp metabolism with immunotherapy, enhancing therapeutic efficacy while mitigating tumor progression, metastasis, and drug resistance. Tailored treatment approaches are essential, considering variations in tumor types and individual circumstances. Despite advancements, the role of Trp metabolism in digestive system tumors requires ongoing research for more effective treatments.

## Data Availability

No datasets were generated or analysed during the current study.

## References

[1] Siegel R, Desantis C, Jemal A (2014). Colorectal cancer statistics, 2014. CA Cancer J Clin..

[2] Cai J, Chen H, Lu M, Zhang Y, Lu B, You L (2021). Advances in the epidemiology of pancreatic cancer: Trends, risk factors, screening, and prognosis. Cancer letters..

[3] Baba Y, Yoshida N, Kinoshita K, Iwatsuki M, Yamashita YI, Chikamoto A (2018). Clinical and Prognostic Features of Patients With Esophageal Cancer and Multiple Primary Cancers: A Retrospective Single-institution Study. Ann Surg.

[4] Patel TH, Cecchini M (2020). Targeted Therapies in Advanced Gastric Cancer. Curr Treat Options Oncol.

[5] Dey P, Kimmelman AC, DePinho RA (2021). Metabolic Codependencies in the Tumor Microenvironment. Cancer Discov.

[6] Biswas SK (2015). Metabolic Reprogramming of Immune Cells in Cancer Progression. Immunity.

[7] Martínez-Reyes I, Chandel NS (2021). Cancer metabolism: looking forward. Nat Rev Cancer.

[8] Yang K, Wang X, Song C, He Z, Wang R, Xu Y (2023). The role of lipid metabolic reprogramming in tumor microenvironment. Theranostics.

[9] Li Z, Zhang H (2016). Reprogramming of glucose, fatty acid and amino acid metabolism for cancer progression. Cellular and molecular life sciences : CMLS.

[10] Solanki S, Sanchez K, Ponnusamy V, Kota V, Bell HN, Cho CS (2023). Dysregulated Amino Acid Sensing Drives Colorectal Cancer Growth and Metabolic Reprogramming Leading to Chemoresistance. Gastroenterol.

[11] Wyant GA, Moslehi J (2022). Expanding the Therapeutic World of Tryptophan Metabolism. Circulation.

[12] Fiore A, Murray PJ (2021). Tryptophan and indole metabolism in immune regulation. Current Opin Immunol..

[13] Cervenka I, Agudelo LZ, Ruas JL (2017). Kynurenines: Tryptophan's metabolites in exercise, inflammation, and mental health. Science..

[14] Castro-Portuguez R, Sutphin GL (2020). Kynurenine pathway, NAD(+) synthesis, and mitochondrial function: Targeting tryptophan metabolism to promote longevity and healthspan. Exp Gerontol..

[15] Liu Y, Pei Z, Pan T, Wang H, Chen W, Lu W (2023). Indole metabolites and colorectal cancer: Gut microbial tryptophan metabolism, host gut microbiome biomarkers, and potential intervention mechanisms. Microbiol Res..

[16] Munn DH, Mellor AL (2016). IDO in the Tumor Microenvironment: Inflammation, Counter-Regulation, and Tolerance. Trends Immunol.

[17] Austin CJ, Rendina LM (2015). Targeting key dioxygenases in tryptophan-kynurenine metabolism for immunomodulation and cancer chemotherapy. Drug Discov Today.

[18] Trézéguet V, Fatrouni H, Merched AJ (2021). Immuno-Metabolic Modulation of Liver Oncogenesis by the Tryptophan Metabolism. Cells..

[19] Santhanam S, Alvarado DM, Ciorba MA (2016). Therapeutic targeting of inflammation and tryptophan metabolism in colon and gastrointestinal cancer. Transl Res.

[20] Su X, Gao Y, Yang R (2022). Gut Microbiota-Derived Tryptophan Metabolites Maintain Gut and Systemic Homeostasis. Cells..

[21] Sun M, Ma N, He T, Johnston LJ, Ma X (2020). Tryptophan (Trp) modulates gut homeostasis via aryl hydrocarbon receptor (AhR). Crit Rev Food Sci Nutr.

[22] Savitz J (2020). The kynurenine pathway: a finger in every pie. Mol Psychiatry.

[23] Kennedy PJ, Cryan JF, Dinan TG, Clarke G (2017). Kynurenine pathway metabolism and the microbiota-gut-brain axis. Neuropharmacol.

[24] Song P, Ramprasath T, Wang H, Zou MH (2017). Abnormal kynurenine pathway of tryptophan catabolism in cardiovascular diseases. Cell Mole Life Sci.

[25] Jovanovic F, Candido KD, Knezevic NN (2020). The Role of the Kynurenine Signaling Pathway in Different Chronic Pain Conditions and Potential Use of Therapeutic Agents. Int J Mol Sci..

[26] Ye Z, Yue L, Shi J, Shao M, Wu T (2019). Role of IDO and TDO in Cancers and Related Diseases and the Therapeutic Implications. J Cancer.

[27] Badawy AA (2017). Kynurenine Pathway of Tryptophan Metabolism: Regulatory and Functional Aspects. Int J Tryptophan Res..

[28] Marszalek-Grabska M, Walczak K, Gawel K, Wicha-Komsta K, Wnorowska S, Wnorowski A (2021). Kynurenine emerges from the shadows - Current knowledge on its fate and function. Pharmacol Ther..

[29] Das YT, Bagchi M, Bagchi D, Preuss HG (2004). Safety of 5-hydroxy-L-tryptophan. Toxicol Lett.

[30] Gibson EL (2018). Tryptophan supplementation and serotonin function: genetic variations in behavioural effects. Proc Nutr Soc.

[31] Dell'Osso L, Carmassi C, Mucci F, Marazziti D (2016). Depression, Serotonin and Tryptophan. Curr Pharm Des.

[32] Maffei ME (2020). 5-Hydroxytryptophan (5-HTP): Natural Occurrence, Analysis, Biosynthesis, Biotechnology, Physiology and Toxicology. Int J Mol Sci..

[33] Balakrishna P, George S, Hatoum H, Mukherjee S (2021). Serotonin Pathway in Cancer. Int J Mol Sci..

[34] Jones LA, Sun EW, Martin AM, Keating DJ (2020). The ever-changing roles of serotonin. Int J Biochem Cell Biol..

[35] Cheng J, Jin H, Hou X, Lv J, Gao X, Zheng G (2017). Disturbed tryptophan metabolism correlating to progression and metastasis of esophageal squamous cell carcinoma. Biochem Biophys Res Commun.

[36] Chen Y, Chen J, Guo D, Yang P, Chen S, Zhao C (2022). Tryptophan Metabolites as Biomarkers for Esophageal Cancer Susceptibility, Metastasis, and Prognosis. Front Oncol..

[37] Zhao Y, Sun J, Li Y, Zhou X, Zhai W, Wu Y (2021). Tryptophan 2,3-dioxygenase 2 controls M2 macrophages polarization to promote esophageal squamous cell carcinoma progression via AKT/GSK3β/IL-8 signaling pathway. Acta pharmaceutica Sinica B.

[38] Pham QT, Oue N, Sekino Y, Yamamoto Y, Shigematsu Y, Sakamoto N (2018). TDO2 Overexpression Is Associated with Cancer Stem Cells and Poor Prognosis in Esophageal Squamous Cell Carcinoma. Oncology.

[39] Kiyozumi Y, Baba Y, Okadome K, Yagi T, Ishimoto T, Iwatsuki M (2019). IDO1 Expression Is Associated With Immune Tolerance and Poor Prognosis in Patients With Surgically Resected Esophageal Cancer. Ann Surg.

[40] Naini AB, Dickerson JW, Brown MM (1988). Preoperative and postoperative levels of plasma protein and amino acid in esophageal and lung cancer patients. Cancer.

[41] Hussain A, Xie L, Deng G, Kang X (2023). Common alterations in plasma free amino acid profiles and gut microbiota-derived tryptophan metabolites of five types of cancer patients. Amino Acids.

[42] Laviano A, Cascino A, Muscaritoli M, Fanfarillo F, Rossi Fanelli F (2003). Tumor-induced changes in host metabolism: a possible role for free tryptophan as a marker of neoplastic disease. Adv Exp Med Biol..

[43] Pham QT, Taniyama D, Akabane S, Takashima T, Maruyama R, Sekino Y (2023). Essential Roles of TDO2 in Gastric Cancer: TDO2 Is Associated with Cancer Progression, Patient Survival, PD-L1 Expression, and Cancer Stem Cells. Pathobiology.

[44] Wu D, Wang Z (2022). Gastric Cancer Cell-Derived Kynurenines Hyperactive Regulatory T Cells to Promote Chemoresistance via the IL-10/STAT3/BCL2 Signaling Pathway. DNA Cell Biol.

[45] Deng K, Lin S, Zhou L, Li Y, Chen M, Wang Y (2012). High levels of aromatic amino acids in gastric juice during the early stages of gastric cancer progression. PLoS ONE.

[46] El-Zaatari M, Bass AJ, Bowlby R, Zhang M, Syu LJ, Yang Y (2018). Indoleamine 2,3-Dioxygenase 1, Increased in Human Gastric Pre-Neoplasia, Promotes Inflammation and Metaplasia in Mice and Is Associated With Type II Hypersensitivity/Autoimmunity. Gastroenterology.

[47] Xiang Z, Li J, Song S, Wang J, Cai W, Hu W (2019). A positive feedback between IDO1 metabolite and COL12A1 via MAPK pathway to promote gastric cancer metastasis. J Exp Clin Cancer Res.

[48] Xu H, Hu C, Wang Y, Shi Y, Yuan L, Xu J (2023). Glutathione peroxidase 2 knockdown suppresses gastric cancer progression and metastasis via regulation of kynurenine metabolism. Oncogene.

[49] Liang X, Du W, Huang L, Xiang L, Pan W, Yang F (2023). Helicobacter pylori promotes gastric intestinal metaplasia through activation of IRF3-mediated kynurenine pathway. Cell Commun Signal.

[50] Shi Z, Gan G, Xu X, Zhang J, Yuan Y, Bi B (2021). Kynurenine derivative 3-HAA is an agonist ligand for transcription factor YY1. J Hematol Oncol.

[51] Zuo X, Chen Z, Cai J, Gao W, Zhang Y, Han G (2019). 5-Hydroxytryptamine Receptor 1D Aggravates Hepatocellular Carcinoma Progression Through FoxO6 in AKT-Dependent and Independent Manners. Hepatology.

[52] Jin H, Zhang Y, You H, Tao X, Wang C, Jin G (2015). Prognostic significance of kynurenine 3-monooxygenase and effects on proliferation, migration, and invasion of human hepatocellular carcinoma. Sci Rep..

[53] Li S, Weng J, Song F, Li L, Xiao C, Yang W (2020). Circular RNA circZNF566 promotes hepatocellular carcinoma progression by sponging miR-4738-3p and regulating TDO2 expression. Cell Death Dis.

[54] Stepien M, Duarte-Salles T, Fedirko V, Floegel A, Barupal DK, Rinaldi S (2016). Alteration of amino acid and biogenic amine metabolism in hepatobiliary cancers: Findings from a prospective cohort study. Int J Cancer.

[55] Li S, Han X, Lyu N, Xie Q, Deng H, Mu L (2018). Mechanism and prognostic value of indoleamine 2,3-dioxygenase 1 expressed in hepatocellular carcinoma. Cancer Sci.

[56] Ishio T, Goto S, Tahara K, Tone S, Kawano K, Kitano S (2004). Immunoactivative role of indoleamine 2,3-dioxygenase in human hepatocellular carcinoma. J Gastroenterol Hepatol.

[57] Li L, Wang T, Li S, Chen Z, Wu J, Cao W (2020). TDO2 Promotes the EMT of Hepatocellular Carcinoma Through Kyn-AhR Pathway. Front Oncol..

[58] Liu P, Zhou Y, Dong X, Zheng B, Liang B, Liang R (2022). ZNF165 Is Involved in the Regulation of Immune Microenvironment and Promoting the Proliferation and Migration of Hepatocellular Carcinoma by AhR/CYP1A1. J Immunol Res..

[59] Yu C, Rao D, Zhu H, Liu Q, Huang W, Zhang L (2021). TDO2 Was Downregulated in Hepatocellular Carcinoma and Inhibited Cell Proliferation by Upregulating the Expression of p21 and p27. BioMed Res Int..

[60] Zor DS, Hakan MT, Özgür E, Horozoglu C, Yörüker EE, Kulle CB (2023). Plasma Levels of Kynurenine, Soluble OX40 and Mir-138-5p Are Associated With Tumor-infiltrating Lymphocytes in Colorectal Cancer: An Exploratory Study. Anticancer Res.

[61] Li T, Fu B, Zhang X, Zhou Y, Yang M, Cao M (2021). Overproduction of Gastrointestinal 5-HT Promotes Colitis-Associated Colorectal Cancer Progression via Enhancing NLRP3 Inflammasome Activation. Cancer Immunol Res.

[62] Sun H, Han W, Wen J, Ma X (2022). IL4I1 and tryptophan metabolites enhance AHR signals to facilitate colorectal cancer progression and immunosuppression. American journal of translational research.

[63] Liu CY, Huang TT, Chen JL, Chu PY, Lee CH, Lee HC (2021). Significance of Kynurenine 3-Monooxygenase Expression in Colorectal Cancer. Front Oncol..

[64] Chen IC, Lee KH, Hsu YH, Wang WR, Chen CM, Cheng YW (2016). Expression Pattern and Clinicopathological Relevance of the Indoleamine 2,3-Dioxygenase 1/Tryptophan 2,3-Dioxygenase Protein in Colorectal Cancer. Dis Markers..

[65] Zhao L, Wang B, Yang C, Lin Y, Zhang Z, Wang S (2021). TDO2 knockdown inhibits colorectal cancer progression via TDO2-KYNU-AhR pathway. Gene..

[66] Ghanipour A, Jirström K, Pontén F, Glimelius B, Påhlman L, Birgisson H (2009). The prognostic significance of tryptophanyl-tRNA synthetase in colorectal cancer. Cancer Epidemiol Biomarkers Prev.

[67] Thaker AI, Rao MS, Bishnupuri KS, Kerr TA, Foster L, Marinshaw JM (2013). IDO1 metabolites activate β-catenin signaling to promote cancer cell proliferation and colon tumorigenesis in mice. Gastroenterology..

[68] Nocito A, Dahm F, Jochum W, Jang JH, Georgiev P, Bader M (2008). Serotonin regulates macrophage-mediated angiogenesis in a mouse model of colon cancer allografts. Can Res.

[69] Park JH, Lee JM, Lee EJ, Kim DJ, Hwang WB (2018). Kynurenine promotes the goblet cell differentiation of HT-29 colon carcinoma cells by modulating Wnt. Notch and AhR signals Oncology reports.

[70] Bishnupuri KS, Alvarado DM, Khouri AN, Shabsovich M, Chen B, Dieckgraefe BK (2019). IDO1 and Kynurenine Pathway Metabolites Activate PI3K-Akt Signaling in the Neoplastic Colon Epithelium to Promote Cancer Cell Proliferation and Inhibit Apoptosis. Can Res.

[71] Miyazaki T, Chung S, Sakai H, Ohata H, Obata Y, Shiokawa D (2022). Stemness and immune evasion conferred by the TDO2-AHR pathway are associated with liver metastasis of colon cancer. Cancer Sci.

[72] Walczak K, Langner E, Szalast K, Makuch-Kocka A, Pożarowski P, Plech T (2020). A Tryptophan Metabolite, 8-Hydroxyquinaldic Acid, Exerts Antiproliferative and Anti-Migratory Effects on Colorectal Cancer Cells. Molecules..

[73] Tomii A, Higa M, Naito K, Kurata K, Kobayashi J, Takei C (2023). Activation of the TLR4-JNK but not the TLR4-ERK pathway induced by indole-3-acetic acid exerts anti-proliferative effects on Caco-2 cells. Biosci Biotechnol Biochem.

[74] Walczak K, Turski WA, Rajtar G (2014). Kynurenic acid inhibits colon cancer proliferation in vitro: effects on signaling pathways. Amino Acids.

[75] Langner E, Walczak K, Jeleniewicz W, Turski WA, Rajtar G (2015). Quinaldic acid inhibits proliferation of colon cancer ht-29 cells in vitro: effects on signaling pathways. Eur J Pharmacol..

[76] León J, Casado J, Jiménez Ruiz SM, Zurita MS, González-Puga C, Rejón JD (2014). Melatonin reduces endothelin-1 expression and secretion in colon cancer cells through the inactivation of FoxO-1 and NF-κβ. J Pineal Res.

[77] Jiang SH, Li J, Dong FY, Yang JY, Liu DJ, Yang XM (2017). Increased Serotonin Signaling Contributes to the Warburg Effect in Pancreatic Tumor Cells Under Metabolic Stress and Promotes Growth of Pancreatic Tumors in Mice. Gastroenterology.

[78] Zhang T, Tan XL, Xu Y, Wang ZZ, Xiao CH, Liu R (2017). Expression and Prognostic Value of Indoleamine 2,3-dioxygenase in Pancreatic Cancer. Chin Med J.

[79] León-Letelier RA, Abdel Sater AH, Chen Y, Park S, Wu R, Irajizad E (2023). Kynureninase Upregulation Is a Prominent Feature of NFR2-Activated Cancers and Is Associated with Tumor Immunosuppression and Poor Prognosis. Cancers (Basel)..

[80] Wang L, Tang W, Yang S, He P, Wang J, Gaedcke J (2020). NO(•) /RUNX3/kynurenine metabolic signaling enhances disease aggressiveness in pancreatic cancer. Int J Cancer.

[81] Hezaveh K, Shinde RS, Klötgen A, Halaby MJ, Lamorte S, Ciudad MT (2022). Tryptophan-derived microbial metabolites activate the aryl hydrocarbon receptor in tumor-associated macrophages to suppress anti-tumor immunity. Immunity.

[82] Huang JY, Butler LM, Midttun Ø, Ulvik A, Wang R, Jin A (2018). A prospective evaluation of serum kynurenine metabolites and risk of pancreatic cancer. PLoS ONE.

[83] Zhang R, Li H, Yu J, Zhao J, Wang X, Wang G (2011). Immunoactivative role of indoleamine 2,3-dioxygenase in gastric cancer cells in vitro. Mol Med Rep.

[84] Walczak K, Dąbrowski W, Langner E, Zgrajka W, Piłat J, Kocki T (2011). Kynurenic acid synthesis and kynurenine aminotransferases expression in colon derived normal and cancer cells. Scand J Gastroenterol.

[85] Brandacher G, Perathoner A, Ladurner R, Schneeberger S, Obrist P, Winkler C (2006). Prognostic value of indoleamine 2,3-dioxygenase expression in colorectal cancer: effect on tumor-infiltrating T cells. Clin Cancer Res.

[86] Ferdinande L, Decaestecker C, Verset L, Mathieu A, Moles Lopez X, Negulescu AM (2012). Clinicopathological significance of indoleamine 2,3-dioxygenase 1 expression in colorectal cancer. Br J Cancer.

[87] Tintelnot J, Xu Y, Lesker TR, Schönlein M, Konczalla L, Giannou AD (2023). Microbiota-derived 3-IAA influences chemotherapy efficacy in pancreatic cancer. Nature.

[88] Witkiewicz A, Williams TK, Cozzitorto J, Durkan B, Showalter SL, Yeo CJ (2008). Expression of indoleamine 2,3-dioxygenase in metastatic pancreatic ductal adenocarcinoma recruits regulatory T cells to avoid immune detection. J Am Coll Surg..

[89] Liu X, Zhou W, Zhang X, Ding Y, Du Q, Hu R (2018). 1-L-MT, an IDO inhibitor, prevented colitis-associated cancer by inducing CDC20 inhibition-mediated mitotic death of colon cancer cells. Int J Cancer.

[90] Alahdal M, Xing Y, Tang T, Liang J (2018). 1-Methyl-D-tryptophan Reduces Tumor CD133(+) cells, Wnt/β-catenin and NF-κβp65 while Enhances Lymphocytes NF-κβ2, STAT3, and STAT4 Pathways in Murine Pancreatic Adenocarcinoma. Sci Rep.

[91] Paccosi S, Cecchi M, Silvano A, Fabbri S, Parenti A (2020). Different effects of tryptophan 2,3-dioxygenase inhibition on SK-Mel-28 and HCT-8 cancer cell lines. J Cancer Res Clin Oncol.

[92] Yang Y, Jin Y, Yin L, Liu P, Zhu L, Gao H (2023). Sertaconazole nitrate targets IDO1 and regulates the MAPK signaling pathway to induce autophagy and apoptosis in CRC cells. Eur J Pharmacol..

[93] Koblish HK, Hansbury MJ, Bowman KJ, Yang G, Neilan CL, Haley PJ (2010). Hydroxyamidine inhibitors of indoleamine-2,3-dioxygenase potently suppress systemic tryptophan catabolism and the growth of IDO-expressing tumors. Mol Cancer Ther.

[94] Yang D, Li T, Li Y, Zhang S, Li W, Liang H (2019). H(2)S suppresses indoleamine 2, 3-dioxygenase 1 and exhibits immunotherapeutic efficacy in murine hepatocellular carcinoma. J Exp Clin Cancer Res.

[95] Liang X, Gao H, Xiao J, Han S, He J, Yuan R (2023). Abrine, an IDO1 inhibitor, suppresses the immune escape and enhances the immunotherapy of anti-PD-1 antibody in hepatocellular carcinoma. Front Immunol..

[96] Korac K, Rajasekaran D, Sniegowski T, Schniers BK, Ibrahim AF, Bhutia YD (2022). Carbidopa, an activator of aryl hydrocarbon receptor, suppresses IDO1 expression in pancreatic cancer and decreases tumor growth. Biochem J.

[97] Jung KH, LoRusso P, Burris H, Gordon M, Bang YJ, Hellmann MD (2019). Phase I Study of the Indoleamine 2,3-Dioxygenase 1 (IDO1) Inhibitor Navoximod (GDC-0919) Administered with PD-L1 Inhibitor (Atezolizumab) in Advanced Solid Tumors. Clin Cancer Res.

[98] Shi D, Wu X, Jian Y, Wang J, Huang C, Mo S (2022). USP14 promotes tryptophan metabolism and immune suppression by stabilizing IDO1 in colorectal cancer. Nat Commun.

[99] Zhang R, Wang Y, Liu D, Luo Q, Du P, Zhang H (2022). Sodium Tanshinone IIA Sulfonate as a Potent IDO1/TDO2 Dual Inhibitor Enhances Anti-PD1 Therapy for Colorectal Cancer in Mice. Front Pharmacol..

[100] Liang H, Li T, Fang X, Xing Z, Zhang S, Shi L (2021). IDO1/TDO dual inhibitor RY103 targets Kyn-AhR pathway and exhibits preclinical efficacy on pancreatic cancer. Cancer Lett..

[101] Sui H, Xu H, Ji Q, Liu X, Zhou L, Song H (2015). 5-hydroxytryptamine receptor (5-HT1DR) promotes colorectal cancer metastasis by regulating Axin1/β-catenin/MMP-7 signaling pathway. Oncotarget.

[102] Schramme F, Crosignani S, Frederix K, Hoffmann D, Pilotte L, Stroobant V (2020). Inhibition of Tryptophan-Dioxygenase Activity Increases the Antitumor Efficacy of Immune Checkpoint Inhibitors. Cancer Immunol Res.

[103] Nozawa H, Taira T, Sonoda H, Sasaki K, Murono K, Emoto S (2023). Enhancement of radiation therapy by indoleamine 2,3 dioxygenase 1 inhibition through multimodal mechanisms. BMC Cancer.

[104] Cao W, Pan J, Mo K, Wang Z, Wei S, Yin Y (2023). Effects of gene silencing of indoleamine 2,3-dioxygenase 1 combined with rosmarinic acid on tumor immune microenvironment in H22 tumor-bearing mice. Int Immunopharmacol..

[105] Chubak J, Boudreau DM, Rulyak SJ, Mandelson MT (2011). Colorectal cancer risk in relation to antidepressant medication use. Int J Cancer.

[106] Sakita JY, Bader M, Santos ES, Garcia SB, Minto SB, Alenina N (2019). Serotonin synthesis protects the mouse colonic crypt from DNA damage and colorectal tumorigenesis. J Pathol.

[107] Huang Q, Xia J, Wang L, Wang X, Ma X, Deng Q (2018). miR-153 suppresses IDO1 expression and enhances CAR T cell immunotherapy. J Hematol Oncol.

